# A New Arterial Variation Involving a Pentafurcated Coeliac Trunk

**DOI:** 10.7759/cureus.26508

**Published:** 2022-07-02

**Authors:** Diego A Abelleyra Lastoria, Alexander Haiser, Vanessa Opoka, David Parry

**Affiliations:** 1 Faculty of Life Sciences and Medicine, King's College London, London, GBR

**Keywords:** embryology to be correlated with gross anatomy, arterial variation, anatomical variability, coeliac trunk, case report

## Abstract

We present a newly found variation of the coeliac trunk. The variation may have clinical implications during surgery and radiological investigations. Open abdominal dissection of an embalmed 65-year-old female cadaver, whose cause of death was metastatic breast carcinoma, was performed in the King’s College London dissection laboratory. Standard, student issue cadaveric dissection equipment was used. A new variation of the coeliac trunk was observed. The variation was a pentafurcated coeliac trunk, with additional arterial variations. The left gastric artery, splenic artery, gastroduodenal artery, middle colic artery, and jejunoileal artery emerged directly from the coeliac trunk. The proper hepatic artery emerged directly from the superior mesenteric artery. The literature review did not reveal the combination of variations that we report. This case reports a new combination of arterial variations. It adds to the knowledge of surgeons and radiologists and highlights the importance of awareness of anatomical variations. Knowledge of anatomical variations may improve patient outcomes.

## Introduction

The coeliac trunk is a ventral branch of the abdominal aorta at the level of T12/L1 vertebrae. Branches of the coeliac trunk are the main blood supply of structures, such as the liver, stomach, and spleen. Branches of the coeliac trunk, in combination with other abdominal aorta branches, supply organs such as the duodenum and pancreas. Normally, the coeliac trunk is approximately 1.25cm in length and consists of three main branches; the left gastric artery, splenic artery, and common hepatic artery [1]. The left gastric artery supplies the lesser curvature of the stomach, where it anastomoses with the right gastric artery. The left gastric artery also gives an artery to the lower part of the esophagus. The splenic artery travels along the superior margin of the pancreas and gives off the short gastric, and pancreatic branches, and the left gastroepiploic artery. The common hepatic artery bifurcates into the proper hepatic artery and the gastroduodenal artery. The proper hepatic artery gives rise to the right and left hepatic arteries and the right gastric artery. The gastroduodenal artery splits into the superior pancreaticoduodenal artery and right gastroepiploic artery. The right gastroepiploic artery anastomoses with the left gastroepiploic artery to supply the greater curvature of the stomach.

Variations from the classically described anatomy of the coeliac trunk have been reported in the literature. One study of 1,569 patients found that in 92.7% of cases, the coeliac trunk gave rise to the left gastric, common hepatic, and splenic arteries. A gastrosplenic trunk (the common origin of the splenic and left gastric arteries) was detected in 4.1%. A hepatosplenic trunk (a common origin of the common hepatic and splenic arteries) was observed in 2.2% of cases. When a hepatosplenic trunk was observed, the left gastric artery arose directly from the abdominal aorta. A coeliaco-mesenteric trunk (a common origin of the coeliac trunk and superior mesenteric artery), was observed in 0.5% of cases [2]. One report described the coeliac trunk as giving rise to the left gastric, common hepatic, splenic, left gastro-epiploic, and right and left inferior phrenic arteries [3]. Another report described the coeliac trunk emerging from the abdominal aorta as two roots, the hepatogastric and hepatosplenic trunk [4].

From a cadaveric dissection, we present a pentafurcated coeliac trunk, with additional arterial variations. The left gastric artery, splenic artery, gastroduodenal artery, middle colic artery, and jejunoileal artery emerged directly from the coeliac trunk. The proper hepatic artery emerged directly from the superior mesenteric artery. The literature review did not reveal the combination of variations that we report.

## Case presentation

All ethical requirements outlined in the Human Tissue Act (2004) regarding the use of cadavers were followed. The dissection was conducted at Kings College London. The cadaver donor was a 65-year-old female. A midline laparotomy incision was made. Bilateral incisions from the midaxillary line, just below the costal margins, joined the midline incision. Bilateral incisions along the line of the inguinal ligaments joined the midline incision. The anterior abdominal wall was reflected, creating an open intraabdominal space. Blunt dissection was used hereon. The abdominal aorta and inferior vena cava were identified. The inferior mesenteric artery, superior mesenteric artery, and coeliac trunk were observed. The coeliac trunk was at the T12 level. All arterial branches were traced using blunt dissection.

**Figure 1:**
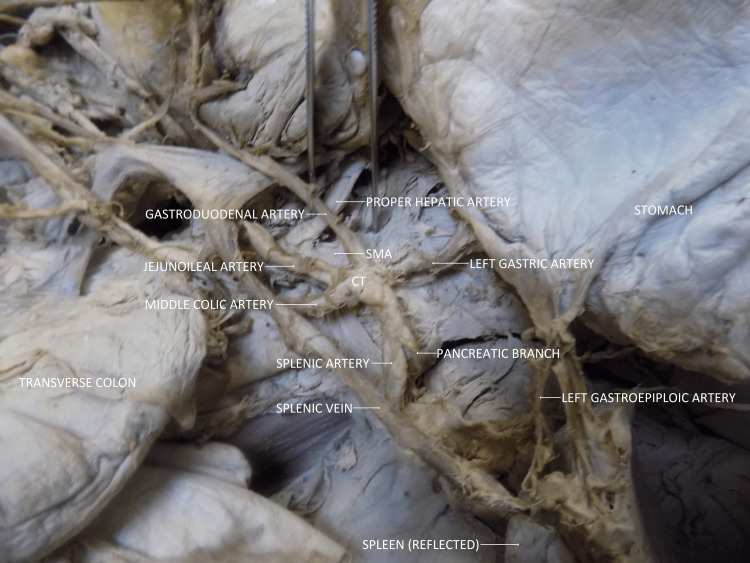
Pentafurcation of the coeliac trunk (CT) as seen in the specimen. The superior mesenteric artery (SMA) gave off the proper hepatic artery.

Our dissection did not show the normal trifurcation of the coeliac trunk into the left gastric artery, common hepatic artery, and splenic artery. Instead, the coeliac trunk pentafurcated into the branches that supply midgut and foregut structures: 1) left gastric artery, 2) splenic artery, 3) gastroduodenal artery, 4) middle colic artery and 5) jejunoileal artery (Figure 1).

The splenic artery was the largest artery and followed a course behind the pancreas. The splenic artery gave two pancreatic branches and the left gastroepiploic artery. The latter travelled along the greater curvature of the stomach. The splenic artery terminated as two branches that entered the splenic hilum.

The left gastric artery travelled along the lesser curvature of the stomach and gave off the inferior esophageal artery. The middle colic artery bifurcated into right and left branches, which supplied the transverse colon. A jejunoileal branch emerged from the coeliac trunk to supply the jejunum and ileum.

The gastroduodenal artery emerged from the coeliac trunk directly. The right gastric artery emerged from the gastroduodenal artery. The gastroduodenal artery then bifurcated into the superior pancreaticoduodenal artery and right gastroepiploic artery. The superior pancreaticoduodenal artery supplied the upper portion of the duodenum. The right gastroepiploic artery continued toward the greater curvature of the stomach and supplied it. An omental branch emerged from the right gastroepiploic artery.

The superior mesenteric artery gave off the proper hepatic artery. The proper hepatic artery gave off the inferior pancreaticoduodenal artery (to supply the inferior duodenum) before becoming part of the portal triad.

## Discussion

This case presents five arteries arising from the coeliac trunk, in comparison to the normally described three arteries. We demonstrate hitherto unreported identifiable arteries originating from the coeliac trunk. The arteries originate from the coeliac trunk as the splenic artery, left gastric artery, gastroduodenal artery, middle colic artery, and jejunoileal artery. In addition to this variation, the proper hepatic artery arose from the superior mesenteric artery, as opposed to its normal origin from the common hepatic artery. Proper hepatic artery arising from the superior mesenteric artery has a reported occurrence in 2%-2.5% of the population [5].

The literature review revealed the presence of multiple variations of branches from the coeliac trunk. A study of 604 selective coeliac and superior mesenteric angiographies identified type I hepatic arterial anatomy (in accordance with the classifications by both Michels and Hiatt) in 79.1% [6]. This corresponds to the common hepatic artery bifurcating into the gastroduodenal artery and proper hepatic artery, with the latter giving rise to the right and left hepatic arteries. An accessory right hepatic artery branching from the superior mesenteric artery was noted in 11.9% of cases [6]. Our case report shows an absent common hepatic artery, with a proper hepatic artery emerging directly from the superior mesenteric artery. One study, using 64-row CT angiography, investigated variations of coeliac trunk branches in 60 patients. A normal trifurcation into the left gastric artery, common hepatic artery, and splenic artery was seen in 56.7% of cases. The common hepatic artery gave rise to its classically described branches (right, left, and proper hepatic arteries and gastroduodenal artery) in 60% of cases [7].

The coeliac trunk and superior mesenteric artery can emerge from the abdominal aorta, combined as a single coeliacomesenteric trunk [8]. One report showed the absence of a coeliac trunk, with the left gastric artery, splenic artery, and common hepatic artery originating directly and separately from the abdominal aorta [9].

Additional variations of the coeliac trunk as summarized in the Bergmann Atlas of anatomical variations include the length of the coeliac trunk and the presence of only two branches (usually the splenic artery and common hepatic artery) or more than three branches [10]. One of the usual branches of the coeliac trunk (left gastric artery, splenic artery, or common hepatic artery) may be replaced by a stem common to the inferior phrenic arteries, by the right gastroepiploic artery, or by the right middle suprarenal artery [10]. The coeliac trunk, superior mesenteric artery, and inferior mesenteric artery may be joined by a longitudinal anastomosis. Additional branches typically include the inferior phrenic artery, middle colic artery, gastroduodenal artery, dorsal pancreatic artery, and jejunal and duodenal branches [11]. A systematic review identified eight forms of variations of the coeliac trunk [12]. The arterial anatomy featured in our report was not identified in any literature, making this coeliac trunk branching pattern a previously unreported anatomical variation.

The variations in this report, are attributable to development. At the end of the third week of gestation, an arterial plexus emerges from the paired dorsal aortae. The arterial plexus connects to the vascular system of the yolk sac and gives rise to the vitelline arteries [13]. The vitelline branches of the fetal abdominal aorta become the blood supply to the gut and are connected by a central longitudinal anastomosis [14]. Three of the vitelline segments arising from the fetal abdominal aorta (10th, 13th, and 21st) persist to form the coeliac trunk, superior mesenteric artery, and inferior mesenteric artery, respectively [15]. These three vessels are formed on day 41 of gestation [13]. Variations of the coeliac trunk and superior mesenteric artery can occur due to the disappearance or survival of the ventral longitudinal anastomosis, between the vitelline branches of the abdominal aorta [14]. Examples of resulting variants include the arc of Buhler (an anastomosis between the coeliac trunk and superior mesenteric artery) and a hepatic artery originating from the superior mesenteric artery [15]. This case report describes a proper hepatic artery originating from the superior mesenteric artery, which corresponds to an incomplete regression of the ventral anastomosis connecting the 10th and 13th vitelline segments [15].

Being aware of anatomical variants is important to prevent iatrogenic injuries during surgery and to improve patient outcomes. Radiologists should be aware of anatomical variations for improving anatomical identification, clinical advising, and interventional treatment. This case presents a hitherto unreported combination of variations that adds new knowledge for surgeons and radiologists.

## Conclusions

Variations of the coeliac trunk are important for abdominal surgeons and radiologists to be aware of. This is particularly relevant when performing operations on foregut and nearby structures or when considering trauma. Radiological awareness of variations enhances clinical outcomes by improving cross discipline advising and interventional procedures. Knowledge of anatomical variations may improve patient outcomes. We present a new combination of arterial variations that will add to the knowledge for surgeons and radiologists.
